# Population demographic history of a temperate shrub, *Rhododendron weyrichii* (Ericaceae), on continental islands of Japan and South Korea

**DOI:** 10.1002/ece3.2576

**Published:** 2016-11-21

**Authors:** Watanabe Yoichi, Ichiro Tamaki, Shota Sakaguchi, Jong‐Suk Song, Shin‐ichi Yamamoto, Nobuhiro Tomaru

**Affiliations:** ^1^ Graduate School of Bioagricultural SciencesNagoya UniversityNagoyaJapan; ^2^ Gifu Academy of Forest Science and CultureMinoGifuJapan; ^3^ Graduate School of Human and Environmental StudiesKyoto UniversityKyotoJapan; ^4^ Department of Biological ScienceCollege of Natural SciencesAndong National UniversityAndongGyeongbukKorea; ^5^ Okayama UniversityOkayamaJapan; ^6^ Present address: Graduate School of HorticultureChiba UniversityMatsudo 648MatsudoChiba271‐8510Japan

**Keywords:** ecological niche modeling, historical vicariance, island biogeography, isolation with migration model, population demography

## Abstract

Continental islands provide opportunities for testing the effects of isolation and migration on genetic variation in plant populations. In characteristic of continental islands is that the geographic connections between these islands, which are currently distinguished by seaways, have experienced fluctuations caused by sea‐level changes due to climate oscillations during the Quaternary. Plant populations on the islands have migrated between these islands via the exposed seafloors or been isolated. Here, we examined the demographic history of a temperate shrub, *Rhododendron weyrichii*, which is distributed in the southwestern parts of the Japanese archipelago and on an island of South Korea, using statistical phylogeographic approaches based on the DNA sequences of two chloroplast and eight nuclear loci in samples analyzed from 18 populations on eight continental islands, and palaeodistribution modeling. Time estimates for four island populations indicate that the durations of vicariance history are different between these populations, and these events have continued since the last glacial or may have predated the last glacial. The constancy or expansion of population sizes on the Japanese islands, and in contrast a bottleneck in population size on the Korean island Jeju, suggests that these islands may have provided different conditions for sustaining populations. The result of palaeodistribution modeling indicates that the longitudinal range of the species as a whole has not changed greatly since the last glacial maximum. These results indicate that exposed seafloors during the glacial period formed both effective and ineffective migration corridors. These findings may shed light on the effects of seafloor exposure on the migration of plants distributed across continental islands.

## 1 Introduction

Continental islands, because they represent isolated distributions of terrestrial habitats, provide suitable settings in which to examine the effects of geographic isolation on evolution in plant species (Bittkau & Comes, 2005; MacArthur & Wilson, 1967; Nakamura, Suwa, Denda, & Yokota, 2009). Continental islands are separated from their adjacent mainlands by shallow seas, which prevent plant populations with low capacities for dispersal from migrating among islands and/or the mainlands, thus leading to increased genetic differentiation. During the late Quaternary, glacial–interglacial climate cycles occurred repeatedly on a global scale (Lisiecki & Raymo, 2005; Petit et al., 1999), and these oscillations have produced drastic changes in the connectivity of isolated populations by reconfiguring the spatial arrangements of islands due to exposure of seafloors that connected continental islands. Spatiotemporal changes in the continuity of species distributions during climatic oscillations could therefore have been a key factor affecting the evolutionary dynamics of species on continental islands by influencing isolation and migration history. In addition, it is important for understanding how the flora of the islands has been shaped.

The Japanese Archipelago, continental islands in East Asia, is a good region for investigating effects of spatiotemporal changes in the continuity of species distributions. It has been inferred that in the Japanese Archipelago, the distribution of temperate forests became shrunken and fragmented across small patches along the coastal regions during glacial periods (Gotanda & Yasuda, 2008; Harrison, Yu, Takahara, & Prentice, 2001; Tsukada, 1985). It is increasingly recognized that the current genetic structure of plant populations has been shaped by descent from populations that sheltered in refugia (Aoki, Suzuki, Hsu, & Murakami, 2004; Iwasaki, Aoki, Seo, & Murakami, 2012; Worth, Sakaguchi, Tanaka, Yamasaki, & Isagi, 2013). However, processes of isolation and migration among refugium populations on islands via exposed seafloor are not clear and have received less attention (Burridge et al., 2013; Duncan, Worth, Jordan, Jones, & Vaillancourt, 2016). In addition, although some studies discussed effects of the last glacial maximum (LGM, Clark et al., 2009) to current genetic variation, these were based on fossil records without focal species, not based on time estimations from genetic data (Iwasaki et al., 2012; Worth et al., 2013).

Understanding the complexity of population demography (population expansion, population decline, divergence, admixture, migration, etc.) on the islands presents several difficulties. Firstly, most studies to date have examined only a few DNA sequences (e.g., regions of the chloroplast DNA [cpDNA] and/or nuclear ribosomal DNA), and stochastic variance between gene genealogies in such a limited number of genomic regions has made it difficult to obtain reliable estimates of population demographies. Secondly, if population genetic analyses are not conducted in the framework of an appropriate time scale, it is not possible to make any biogeographically meaningful inferences about the drivers of population divergence. This is particularly problematic in phylogeographic analyses of continental island species, because here population divergence can be triggered by interglacial geographic isolation and/or glacial habitat isolation. In the last decade, demographic modeling of natural populations based on the coalescent approach has become commonplace (Hey & Nielsen, 2004; Nielsen & Beaumont, 2009). This approach can statistically estimate demographic parameters such as migration rate, divergence time, and changes in population size within a given time scale. Multilocus DNA sequences offer the potential for inferring species’ demographic histories in great detail because they provide many genetically informative segregating sites and independent genealogies (Wang et al., 2014). Ecological niche modeling can also provide useful information about historical range shifts by reconstructing palaeodistributions during key periods, for example, the LGM. These approaches enable us to integrate phylogeographic inferences and information about spatial range shifts, and as a result, they can lead to deeper insights into species’ biogeographic histories during the late Quaternary (Phillips, Anderson, & Schapire, 2006; Svenning, Fløjgaard, Marske, Nógues‐Bravo, & Normand, 2011).

**Figure 1:**
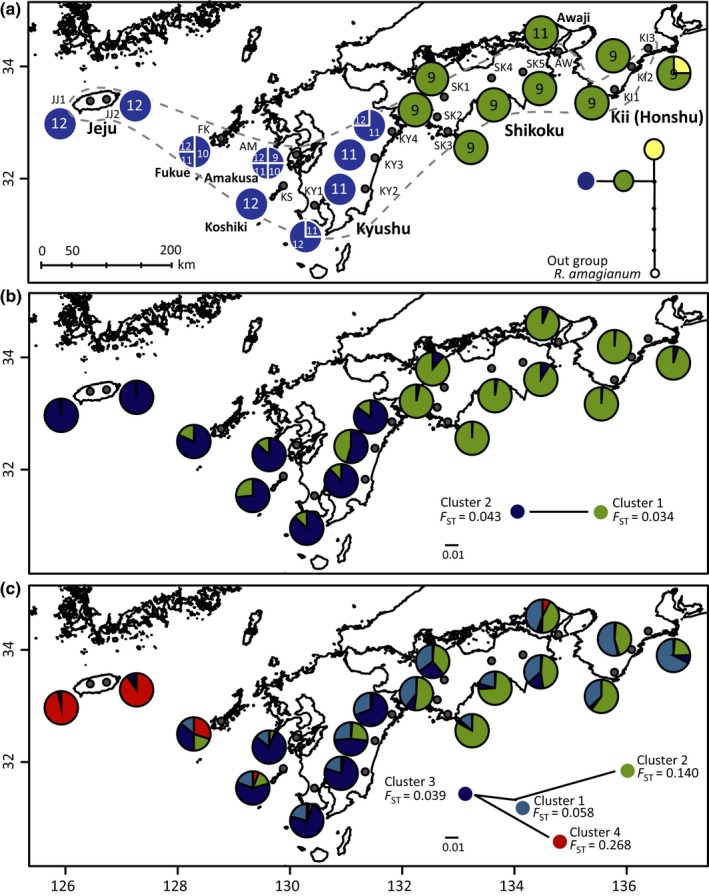
Species range and geographic distribution of (a) three haplotypes, showing the number of tri‐nucleotide repeats (ATT) at chloroplast DNA loci and (b) two and (c) four genetic clusters detected by STRUCTURE analysis based on haplotypes at nuclear DNA loci in *Rhododendron weyrichii* populations. (a) Small thin letters indicate population codes corresponding to those in Table 1; numbers in pie charts indicate the numbers of tri‐nucleotide repeats; a parsimony network superimposed on the map shows the relationships among the haplotypes using *Rhododendron amagianum* as an out‐group. (b) and (c) A neighbor‐joining tree superimposed on each map shows the relationships among the clusters based on net nucleotide distances; *F*_ST_ of each cluster indicates the extent of genetic divergence from the ancestral population

*Rhododendron weyrichii* Maxim. (Ericaceae) is a deciduous shrub species, occurring mainly in temperate regions that experience high rainfall during summer in southwestern parts of the Japanese Archipelago including the Kii Peninsula of Honshu, Shikoku, and Kyushu islands and small islands surrounding them, and in Jeju Island in the southwest of the Korean Peninsula, disjunctly (Figure 1; Chamberlain & Rae, 1990). These regions are thought to have been important glacial refugia for temperate plant species (Gotanda & Yasuda, 2008; Harrison et al., 2001). *Rhododendron weyrichii* produces numerous small seeds (30–40 mg/100 seeds; Yoichi W., personal observation). Its pollinators are butterflies and bumblebees, and thus, exchange of pollen between island populations is infrequent under present‐day interglacial conditions. However, the dispersal barriers formed by seaways among neighboring islands disappeared during the LGM (ca. 21 kya) due to the shallow depth of the sea (<120 m, Clark et al., 2009; Park, Kimura, & Taira, 1996; Siddall et al., 2003), and this could have opened up effective dispersal corridors for the species. As *R. weyrichii* is widespread across a number of islands and shows a moderate level of genetic differentiation among populations (Yoichi & Tomaru, 2014), and it provides a suitable study system for analyzing demographic history in the islands.

The configuration of the landscape in these island regions is believed to have affected the distribution and demographic history of plant populations. In this study, we examined the demographic history of *R. weyrichii* populations using statistical phylogeographic approaches based on chloroplast and nuclear DNA (nDNA) sequences. We also examined the distribution of the species during the LGM using ecological niche modeling. The specific aims of the study were (1) to estimate population genetic divergence between continental islands and to address the isolation–migration histories of these populations; (2) to evaluate differences in genetic diversity and historical changes in population size among islands; and (3) to make inferences about population survival on each island during the LGM.

## 2 Materials and Methods

### 2.1 Plant material

Leaf samples were collected from 142 *R. weyrichii* individuals from 18 populations on eight continental islands (Figure 1, Table 1). Thirty‐two individuals from four populations of *Rhododendron sanctum* and one individual of *Rhododendron amagianum* were used as out‐groups in, respectively, nDNA and cpDNA analyses. Voucher specimens from each population were deposited in the National Museum of Nature and Science, Japan (TNS).

**Table 1:** Locality and the numbers of individuals used in chloroplast and nuclear DNA analyses and genetic diversity and neutrality statistics over eight nuclear loci, for 18 populations from eight continental islands

Continental island	Population code and locality		Coordinate	*n*_c_	*n*_n_	π	θ_W_	*D*
Jeju Island	JJ1	Eorimok, Jeju, South Korea	33.3883N/126.4954E	4	16	0.0042	0.0032	0.656
	JJ2	Saryoni, Jeju, South Korea	33.4180N/126.6353E	4	16	0.0036	0.0037	−0.262
Fukue Island	FK	Mt. Sasa, Nagasaki, Japan	32.7216N/128.8099E	4	16	0.0042	0.0039	0.092
Amakusa Island	AM	Mt. Kado, Kumamoto, Japan	32.3997N/130.0965E	4	16	0.0041	0.0046	−0.544
Koshiki Island	KS	Segami, Kagoshima, Japan	31.8618N/129.8698E	4	16	0.0043	0.0040	0.156
Kyushu Island	KY1	Mt. Kumagadake, Kagoshima, Japan	31.4551N/130.4672E	4	16	0.0046	0.0046	0.062
	KY2	Mt. Boroishi, Miyazaki, Japan	31.8039N/131.3800E	4	16	0.0045	0.0052	−0.439
	KY3	Mt. Kanmuri, Miyazaki, Japan	32.3849N/131.5385E	4	16	0.0042	0.0047	−0.370
	KY4	Kitaura, Miyazaki, Japan	32.7336N/131.8325E	4	16	0.0054	0.0057	−0.362
Shikoku Island	SK1	Kawabe, Ehime, Japan	33.5020N/132.7661E	4	14	0.0035	0.0041	−0.387
	SK2	Mt. Okubo, Kochi, Japan	33.1855N/132.6120E	4	16	0.0042	0.0043	−0.166
	SK3	Mt. Imano, Kochi, Japan	32.8590N/132.8537E	4	16	0.0024	0.0027	−0.271
	SK4	Reihoku, Kochi, Japan	33.7021N/133.6039E	4	16	0.0036	0.0034	0.062
	SK5	Mt. Syozanji, Tokushima, Japan	33.9842N/134.3040E	4	16	0.0023	0.0029	−0.571
Awaji Island	AW	Mt. Yuzuriha, Hyogo, Japan	34.2435N/134.8358E	4	16	0.0037	0.0037	−0.076
Kii Peninsula of	KI1	Mt. Kasane, Wakayama, Japan	33.5218N/135.8042E	4	16	0.0038	0.0045	−0.475
Honshu Island	KI2	Siro, Mie, Japan	33.9644N/136.1933E	4	14	0.0036	0.0044	−0.321
	KI3	Nisaka, Mie, Japan	34.2359N/136.3587E	4	16	0.0044	0.0046	−0.040

*n*_c_ and *n*_n_, number of alleles used in chloroplast and nuclear DNA analyses, respectively; π, nucleotide diversity; θ_W_, Watterson's θ per site; *D*, Tajima's *D*.

Population codes correspond to those in Figure 1.

### 2.2 DNA extraction and sequencing

Genomic DNA was extracted from leaf samples using the cetyltrimethylammonium bromide method (Murray & Thompson, 1980), after treatment with sorbitol extraction buffer (Wagner et al., 1987). Two cpDNA and eight nDNA regions were sequenced in order to detect DNA polymorphisms. Two cpDNA loci (*trnG* intron and *rpl36*‐*rps8*) were PCR‐amplified from four individuals per population using universal primers (Kress, Wurdack, Zimmer, Weigt, & Janzen, 2005; Shaw et al., 2005). For the eight nDNA loci, primers developed for other Ericaceous species (C16 and C22 [Wei, Fu, & Arora, 2005], EST39, EST65, EST121, and EST136 [De Keyser, De Riek, & Van Bockstaele, 2009], PHYB and PHYE [Ikeda & Setoguchi, 2010]) were used for PCR amplification of seven or eight individuals per population (Table S1). PCR was performed with an initial denaturation for 4 min at 94°C followed by 35 cycles of denaturation for 60 s at 94°C, annealing for 60 s at 55 or 60°C and extension for 60 s at 72°C, and a final extension for 7 min at 72°C, using AmpliTaq Gold Master Mix (Applied Biosystems). After PEG precipitation (Hartley & Bowen, 1996), the PCR products were sequenced directly using the standard method provided with the BigDye Terminator Cycle Sequencing kit v. 3.1 (Applied Biosystems) and separated by electrophoresis on an ABI 3100 Genetic Analyzer (Applied Biosystems).

### 2.3 Data analysis of chloroplast DNA sequences

The cpDNA sequences acquired were assembled using DNA Baser v. 3 (Heracle BioSoft S.R.L.) and aligned using the Muscle algorithm implemented in MEGA v. 5 (Edgar, 2004; Tamura et al., 2011). Mononucleotide repeats in the sequences were omitted from subsequent analyses to avoid the possibility of homoplasy. The number of tri‐nucleotide repeats ([ATT]_n_) found in *rpl36‐rps8* was counted, but these repeats were excluded from alignments when determining cpDNA haplotypes. Genealogical relationships for all cpDNA haplotypes were constructed as a parsimony network using TCS v. 1.06 (Clement, Posada, & Crandall, 2000).

### 2.4 Data analysis of nuclear DNA sequences

#### 2.4.1 Estimation of genetic diversity and genetic structure

The nDNA sequences acquired were also assembled using DNA Baser and aligned using Muscle in MEGA; indel polymorphisms in these sequences were omitted from subsequent analyses. The haplotype phases of the aligned sequences were determined using PHASE v. 2.1 (Stephens & Scheet, 2005; Stephens, Smith, & Donnelly, 2001) implemented in DnaSP v. 5 (Librado & Rozas, 2009). Three independent runs employing the general model for recombination rate variation were conducted with a burn‐in period of 1,000 followed by 10,000 iterations with a thinning interval of 100 steps. Those haplotypes with a posterior probability of >0.90 were used in subsequent analyses. Homology search using blastx was conducted against polypeptide sequences from the NCBI and *Arabidopsis thaliana* (TAIR; http://www.arabidopsis.org) databases to identify a possible function for the product of each locus.

Number of segregating sites (*S*) and nucleotide diversity (π; Nei, 1987) were calculated for each locus. The minimum number of recombinations (*R*_M_) at each locus was estimated based on the four‐gamete test (Hudson & Kaplan, 1985). Neutrality at each locus was tested using Tajima's *D* (Tajima, 1989) and Fay and Wu's *H* (Fay & Wu, 2000). Fay and Wu's *H* was tested with out‐group (*R. sanctum*) sequences, and significance was determined using 10,000 coalescent simulations. In addition, an HKA test (Hudson, Kreitman, & Aguadé, 1987) was conducted to test for neutral evolution across loci between species (*R. weyrichii* and *R. sanctum*). All of these analyses were performed using DnaSP, with the exception of the HKA test which was carried out using the HKA program (http://genfaculty.rutgers.edu/hey/software#HKA). Within‐population genetic diversity was evaluated for each population. Nucleotide diversity, Watterson's θ (Watterson, 1975), and Tajima's *D* were calculated over all loci within each population using DnaSP.™

Population genetic structure was then estimated as follows. The average number of nucleotide differences (*D*_*xy*_; Nei, 1987) over all loci between populations was calculated. Genealogical relationships among all haplotypes at each locus were estimated by constructing a parsimony network using TCS. Population genetic structure based on genotypes (two haplotypes) at each locus for each individual was estimated by model‐based Bayesian clustering analysis using STRUCTURE v. 2.3.3 (Pritchard, Stephens, & Donnelly, 2000). Admixture, correlated allele frequency and LOCPRIOR models were used (Falush, Stephens, & Pritchard, 2003; Hubisz, Falush, Stephens, & Pritchard, 2009). MCMC simulations were performed with 20 independent runs for each cluster (*K *=* *1–10), with 3.0 × 10^4^ iterations after a burn‐in period of 2.0 × 10^4^. The optimal value of *K* was estimated by plotting the log likelihood of the data, Ln Pr(*X*|*K*) (Pritchard et al., 2000), and the Δ*K* statistic, which was calculated from the second‐order rates of changes of Ln Pr(*X*|*K*) (Evanno, Regnaut, & Goudet, 2005), against *K*. A neighbor‐joining among‐clusters tree was constructed based on the net nucleotide distances between pairs of clusters, and *F*_ST_ values between the ancestral population and each cluster, which represent the extent of genetic drift undergone by each cluster after differentiation from the ancestral population, were calculated (Pritchard et al., 2000).

#### 2.4.2 Demographic analysis

Demographic history was firstly inferred using the isolation with migration (IM) model. To estimate the IM history among four major regional population groups (Jeju, Kyushu, Shikoku, and Kii), the population demographic parameters (Figure 3) population split time (*t *=* T*μ), which is a product of the absolute time in years (*T*) and the mutation rate (μ), population size (θ = 4*N*μ, where *N* is effective population size), migration rate per mutation event (*m *= *M*/μ, where *M* is migration rate), and population migration rate (2*NM* = 0.5θ*m*) were estimated using IMa2 (Hey & Nielsen, 2004, 2007; see Appendix S1).

The approximate Bayesian computation (ABC) approach enables us to infer demographic history by estimating the values of parameters in a complex model without calculating its likelihood (Beaumont, 2010; Csillery, Blum, Gaggiotti, & Francois, 2010). In this study, four different demographic models related to changes in population size (Fig. S3), a standard neutral model (Model 1), an exponential growth model (Model 2), an instantaneous size reduction model (Model 3), and an exponential growth after instantaneous size reduction model (Model 2 + Model 3, Model 4) were examined for each of the four island populations using R v. 3.2.1, ms and msABC (Hudson, 2002; Pavlidis, Laurent, & Stephan, 2010; R Core Team, 2015; see Appendix S1).

### 2.5 Ecological niche modeling

To infer changes in distribution between the LGM and the present, we produced distribution models for *R. weyrichii* based on modern distribution records and bioclimatic variables using maximum entropy methods (Phillips et al., 2006). Species occurrence data were obtained from the 5th Specific Plant Community Survey (http://www.biodic.go.jp/reports2/5th/vgt_toku/index.html), specimens in two herbaria (the Herbarium of the University of Tokyo and the National Science Museum, Tsukuba), and personal observations. In total, 172 presence records for *R. weyrichii* were obtained covering the entire range of the species, after removal of duplicate records from within each 2.5‐arc‐minutes cell. Current climate data at a resolution of 2.5 arc‐minutes were obtained from WorldClim (Hijmans, Cameron, Parra, Jones, & Jarvis, 2005). Validation of the distribution model was performed, using default settings with 100 replicates of cross‐validation procedures, with 25% of the data used for model testing, implemented in Maxent v. 3.3.3e (Phillips et al., 2006). Five bioclimatic variables (annual mean temperature, mean temperature in the driest quarter, annual precipitation, precipitation in the wettest quarter, and precipitation in the driest quarter), which showed high values of area under curve (AUC) and are critically important for temperate species that are distributed in humid and warm environments, were chosen for inclusion in the distribution model, as they have been used in ecological niche modeling for other species with similar distribution ranges (Qi, Yuan, Comes, Sakaguchi, & Qiu, 2014; Worth et al., 2013).

The model calibrated with current climate conditions was projected onto the reconstructed climate during the LGM obtained from two climatic simulations, the Community Climate System Model (CCSM; Collins et al., 2006) and the Model for Interdisciplinary Research on Climate (MIROC; Hasumi & Emori, 2004), obtained from the PMIP2 website (http://pmip2.lsce.ipsl.fr/). We prepared the LGM palaeoclimate layers at a resolution of 2.5 arc‐minutes following the methods of Sakaguchi et al. (2012), and the palaeocoastlines were estimated as being −130 m lower than at present on the basis of seafloor topology data (ETOPO1; http://www.ngdc.noaa.gov/mgg/global/).

## 3 Results

### 3.1 Genetic diversity and structure of chloroplast DNA

The sequences of two cpDNA loci were obtained from 72 individuals across 18 populations of *R. weyrichii* (Table 1). The lengths of aligned sequences for the two loci were 557 bp in *trnG* intron and 455 bp in *rpl36*‐*rps8*. There were three haplotypes with three segregating sites across the two loci (Figure 1a). Tri‐nucleotide repeats (ATT) that were identified in a *rpl36*‐*rps8* intergenic spacer showed a high level of variation in Kyushu. The geographic distribution of haplotypes showed a clear division between Shikoku and Kyushu, and a distinct haplotype was recognized in a population from Kii.

### 3.2 Genetic diversity and neutrality of nuclear DNA

The sequences of eight nDNA loci were obtained from 142 individuals across 18 populations (Table 1). The length of aligned sequences for each locus ranged from 339 bp for PHYE to 470 bp for EST136 (Table 2), and the total length was 3,340 bp. The eight nDNA loci showed a high degree of polymorphism, with the number of segregating sites (*S*) ranging from 9 to 25 and the nucleotide diversity for all sites (π) ranging from 0.0018 to 0.0060. Recombination events were recognized for seven loci, all except PHYE, and the number of minimum recombination events (*R*_M_) ranged from 0 to 9 (Table 2). In the two neutrality tests, which examine intraspecific variation (Tajima's *D* test) and interspecific (i.e., between *R. weyrichii* and *R. sanctum*) variation (Fay and Wu's *H* test), neutral evolution of every locus was not rejected (Table 2). In addition, the HKA test did not reject neutrality for the divergence between *R. weyrichii* and *R. sanctum* (χ^2^ = 15.043, *p *=* *.375). The π, Watterson's θ (θ_W_), and *D* values for each population ranged from 0.0024 to 0.0054, 0.0027 to 0.0057, and −0.571 to 0.656, respectively (Table 1).

**Table 2:** Genetic diversity and neutrality statistics over eight nuclear DNA loci across all the sampled populations

Locus	Alignment length (bp)	Largest non‐recombining block (bp)	*N*	*S*	*h*	*R*_M_	π	*D*	*H*
EST39	461	394	278	25	55	9	0.0060	−0.984	1.107
EST65	387	382	276	18	38	4	0.0027	−0.415	0.289
EST121	387	380	270	21	28	3	0.0052	−1.085	−2.202
EST136	470	431	272	24	55	5	0.0043	−1.353	−0.514
C16	407	389	246	24	30	3	0.0053	−1.286	1.765
C22	425	388	286	9	13	2	0.0037	0.202	−1.573
PHYB	464	464	274	12	18	2	0.0018	−1.462	0.741
PHYE	339	339	286	9	9	0	0.0039	−0.198	−0.858

*N*, number of alleles sequenced; *S*, number of segregating sites; *h*, number of phased haplotypes detected; *R*_M_, minimum number of recombination events; π, nucleotide diversity; *D*, Tajima's *D*;* H*, Fay and Wu's *H*.

Tajima's *D* and Fay and Wu's *H* were not significant at any locus.

### 3.3 Population genetic structure of nuclear DNA

**Figure 2:**
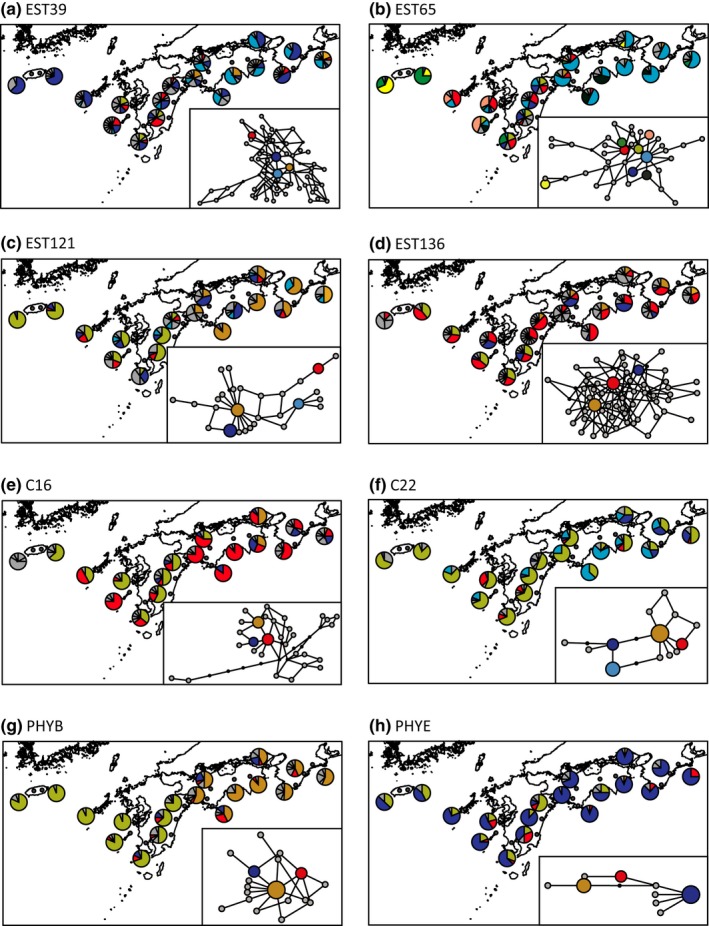
Geographic distributions of haplotypes at nine nuclear loci: (a) EST39, (b) EST65, (c)EST121, (d) EST136, (e) C16, (f) C22, (g) PHYB, and (h) PHYE. A parsimony network superimposed on each map shows the relationships among haplotypes at each locus

The pairwise genetic divergence (the average number of nucleotide differences, *D*_*xy*_) between populations ranged from 0.0031 to 0.0069 (Table S2). Each nuclear locus had major haplotypes, and variable patterns of haplotype distribution were detected among loci (Figure 2). In the Bayesian clustering analysis, *K *=* *2 was the optimal number of clusters supported by the Δ*K* statistics, and *K *=* *4 was the optimal number of clusters that showed a plateau in the log‐likelihood value and low variance among runs (Fig. S1). In the case of *K *=* *2, the distribution of clusters showed a division between Kyushu and Shikoku (Figure 1b). In the case of *K *=* *4, clusters 1 and 2 were recognized in the populations of the Japanese Archipelago, cluster 3 was recognized mainly in Kyushu and the adjacent islands (Koshiki, Amakusa, and Fukue), and cluster 4 predominated in Jeju and was also recognized in Fukue (Figure 1c). The *F*_ST_ values for clusters 2 and 4 showed higher values than those for clusters 1 and 3; in addition, clusters 2 and 4 were distant from clusters 1 and 3. Clear genetic divergence between Jeju–Kyushu and Shikoku–Kii was also supported by the phylogenetic relationships between island populations (Fig. S2).

### 3.4 Demographic history of four island populations

**Figure 3:**
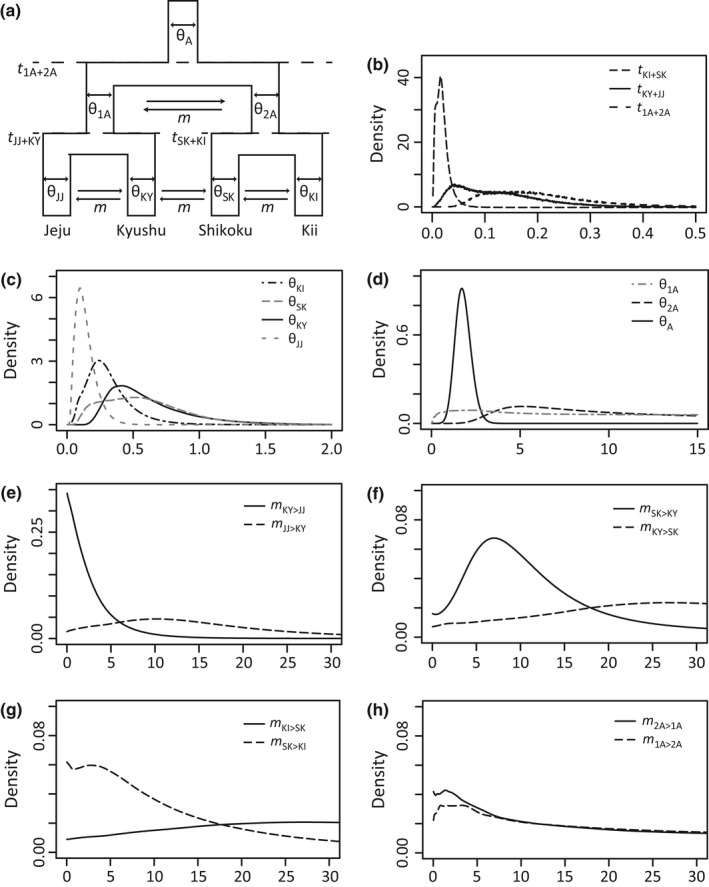
(a) An isolation with migration (IM) model for the four island populations examined in this study (above left), and the marginal distribution of posterior probabilities for (b) the split time (*t*), (c, d) population size (θ), and (e–h) migration rate between populations (*m*)

The posterior modes and 95% highest posterior density (HPD) intervals for the split time (*t*) between Jeju and Kyushu (*t*_JJ+KY_), between Shikoku and Kii (*t*_SK+KI_), and between Jeju–Kyushu and Shikoku–Kii (*t*_1A+2A_) estimated by the IM model were 0.043 (0.008–0.276), 0.006 (0.001–0.041), and 0.151 (0.051–0.384), and the corresponding estimates of the divergence time (*T*) between these islands were 69 kya (13–443), 9 kya (2–65), and 243 kya (81–616), respectively (Figure 3, Table S3). The nested models, which hypothesized zero migration (i.e., the migration rates [*m*] and population migrations [2*NM*] were set on the assumptions of a zero migration hypothesis [*m* and 2*NM *= 0]), were not rejected by likelihood ratio tests, with the exceptions of migration from Shikoku to Kyushu and from Kyushu to Jeju in the sense of time forward.

**Figure 4:**
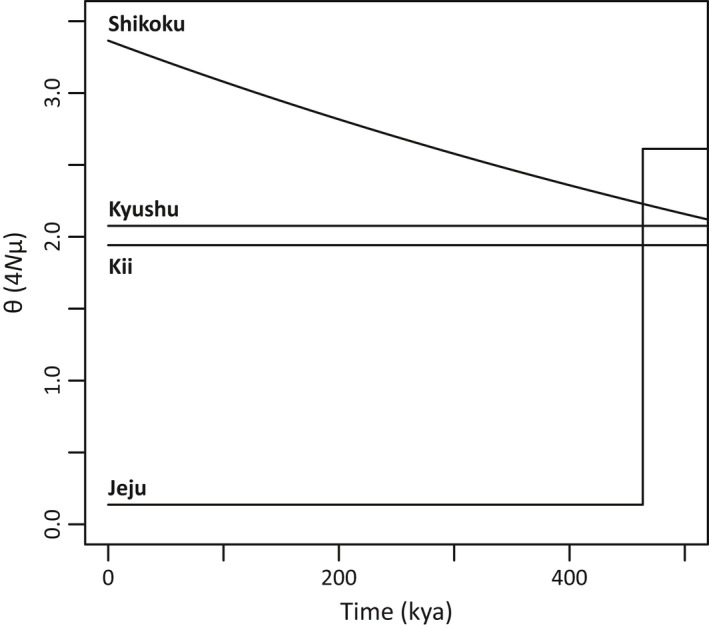
Historical changes in population size (θ) for the populations on four continental islands over time. θ = 4*N*μ, where *N* is effective population size and μ is mutation rate. Time is on a scale of thousands of years ago, kya

In the ABC analysis of each island population, the standard neutral model (Model 1) was supported for Kii and Kyushu, whereas the exponential growth model (Model 2) was supported for Shikoku and the instantaneous size reduction model (Model 3) was supported in the case of Jeju (Table 3, Figs S3, S4) with good agreement between the posterior predictive simulations for the observed and simulated data sets (Fig. S5). It was estimated that expansion or maintenance of population size in Kii, Shikoku, and Kyushu continued over the last glacial period (Figure 4). The Jeju population exhibited a significantly lower value for population size (mode of θ_0_ [95% HPD] = 0.136 [0.000–0.673]) than the other populations (Figure 4, Table S4). In addition, a bottleneck event in Jeju was estimated to have happened before the last glacial (467 kya [184–1,026]), and the effective population size has been low since then. When comparing the results of IM and ABC analyses, although the values of θ_0_ for each island in the ABC analysis showed higher values than those in IM analysis, the relative relationship of θ_0_ among islands was the same in both analyses, including the finding that the lowest value was that for Jeju.

**Table 3:** Posterior probabilities of four demographic models for the populations on four continental islands

Population	Demographic model^a^			
	Model 1	Model 2	Model 3	Model 4
Jeju	0.000	0.000	**0.817**	0.183
Kyushu	**0.547**	0.158	0.145	0.150
Shikoku	0.112	**0.550**	0.025	0.313
Kii	**0.488**	0.188	0.149	0.176

The models accepted are shown in boldface.

a Model 1, standard neutral model; Model 2, exponential growth model; Model 3, instantaneous size reduction model; Model 4, exponential growth after instantaneous size reduction model.

### 3.5 Differences in species distribution between the last glacial maximum and the present time

**Figure 5:**
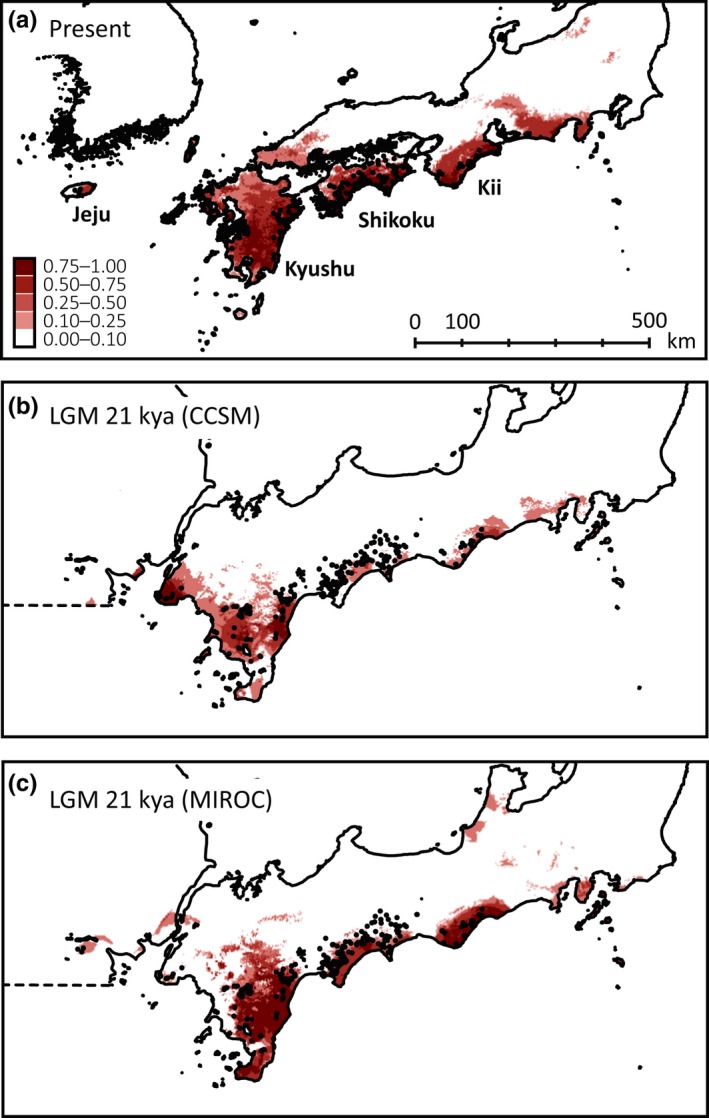
Predicted distribution of *Rhododendron weyrichii* (a) at present, and at the last glacial maximum (LGM, 21 thousand years ago) under two climatic models (b) the Community Climate System Model, CCSM, and (c) the Model for Interdisciplinary Research on Climate, MIROC. The range of red color intensity indicates the logistic distribution probability, and locations of the present distribution are plotted as black points on the maps

The Maxent model showed a relatively high accuracy (area under curve, AUC = 0.940 ± 0.009). The predicted distribution based on current climatic conditions was similar to the actual distribution of the species with high probability (>0.50), although some areas along the Pacific Ocean side and on the western edge of Honshu were also predicted to be suitable (Figure 5a). The contribution made by environmental variables to the model prediction was higher for precipitation (annual precipitation = 71.8%, precipitation in the driest month = 12.0%) than for temperature (annual mean temperature = 3.8%, mean temperature in the driest season = 6.0%). Under the two LGM climate simulations (CCSM and MIROC), it was estimated that suitable environments had been maintained on three of the four islands (Figure 5b, c), but the areas on the map with high probabilities (>50%) had shrunk. In addition, the area with a suitable environment on Jeju showed low probabilities and restricted distributions in both models. It is noteworthy that although the exposed seafloor physically connected the major islands during the LGM, the ranges suitable for the species were somewhat scattered between neighboring islands.

## 4 Discussion

### 4.1 Isolation and migration among island populations during the late Quaternary

The estimates of the times of divergence between *R. weyrichii* populations on these four islands indicate that timing and duration of population divergence is different. In particular, the clear genetic divergence between Jeju–Kyushu and Shikoku–Kii was supported by the genetic structure given by both nuclear and chloroplast DNA sequences, and the time estimate suggests that the vicariance may have continued since the last glacial or have predated the last glacial (Petit et al., 1999). While, the most recent divergence between Shikoku and Kii may indicate a role of exposed seafloor for migration corridor. However, because time estimates are seriously influenced by mutation rate, and by variations in mutation rate among sites depending, for example, on whether substitutions are synonymous or nonsynonymous (Wolf, Li, & Sharp, 1987), careful consideration is necessary when interpreting population history.

In addition, the results of a likelihood ratio test of the IM analysis suggest that a few migration events among island populations happened after their divergence. The distribution of genetic clusters detected by STRUCTURE analysis indicates that Fukue, which is located midway between Jeju and Kyushu, may have been a region of land bridges that were used like stepping stones. These evidences may mean that the exposed seafloors during the last glacial have acted as both effective and ineffective migration corridors between islands (Siddall et al., 2003).

### 4.2 Differences in population survival among islands

The three island populations in the Japanese Archipelago (those on Kyushu, Shikoku, and Kii) showed large population sizes (θ_0_) than those on Jeju according to ABC analysis; the pattern was similar for the result of IM analysis. Palynological studies have suggested that warm‐temperate forests may have retreated to southern refugia and been replaced by cold‐tolerant coniferous and broadleaf vegetation throughout most of the Japanese Archipelago during the LGM (Gotanda & Yasuda, 2008). In addition, previous genetic studies on many warm‐temperate species have indicated that southwestern parts of the Japanese Archipelago provided important refugia (Aoki et al., 2004; Zhai, Comes, Nakamura, Yan, & Qiu, 2012). These results suggest that climate oscillations had negligible impacts at the southern tips of the merged “Japanese island” at that time, and the populations may therefore have been maintained in situ in each region throughout at least one cycle of climate change (Clark et al., 2009). Another minor factor that affects genetic diversity and population size of certain species is introgression from related species. A chloroplast haplotype unique among *R. weyrichii* was recognized in the north of Kii. This haplotype was shared with *R. kiyosumense* Makino (Yoichi unpublished data), which is distributed on the Pacific Ocean side of Honshu. An intraspecific taxon with a different flower color, which is recognized as *R. weyrichii* f. *purpureum* Hara, in the northern part of Kii is considered to have resulted from introgression events from the other species, as has also occurred in other closely related *Rhododendron* species (Morimoto et al., 2005; Tagane, Hiramatsu, & Okubo, 2008).

In contrast to the populations in the Japanese Archipelago, the estimated value of effective population size on Jeju was low, and this is likely to have been caused not by a recent bottleneck event. In addition, the effective population size on Jeju has not expanded after the bottleneck event. Although the time estimates given by IM and ABC analyses exhibited different values, this difference may be the result of an estimation error due to difference in examined models and model simplification. At least the accepted model and the time estimate suggested that the Jeju population is not likely to have been formed by recent migration from the Japanese island. The Jeju population is currently restricted along rivers in warm temperate forests characterized by trees of *Carpinus* or *Quercus* whereas fossil pollen records suggest that the island has been dominated by grassland with patches of cool‐temperate deciduous broadleaved woodland and that conditions were dry during the LGM (Chung, 2007; Lee, Lee, Yoon, & Yoo, 2008). In addition, ecological niche modeling predicted that relatively small areas of the Jeju region were suitable, and the probability values for the distribution were not high. These lines of evidences indicated that Jeju was not suitable for *R. weyrichii*; however, Bayesian clustering and demographic analyses suggested that *R. weyrichii* may have survived in restricted and scattered habitats on or around the island during the LGM. The possibility of the invisible refugia is supported by the high genetic diversity of tree species on the island (Chung et al., 2013; Lee, Lee, & Choi, 2013; Lee, Lee, Choi, & Choi, 2014). These regional differences in population size between the islands suggest that environmental sustainability is a key factor in maintaining a population under conditions in which there are few migrations among populations (Frankham, 2005; Morjan & Rieseberg, 2004).

### 4.3 Survivals of island populations from the last glacial maximum up to the present

When considering isolation and migration among islands, the LGM is an important period in history (Hewitt, 2000, 2004). Under both the CCSM and the MIROC simulation, suitable areas were predicted on each of the four islands, but these areas appeared to lack spatial connections across the exposed seafloor. The existence of at least possible two regions on the islands containing refugia during the LGM, Kii–Shikoku and Kyushu–Jeju, was also supported by IM analysis. Thus, the latitudinal and/or longitudinal range of the species may not have changed greatly since the LGM as is also the case for other forest species on continental islands (Duncan et al., 2016; Qi et al., 2014; Qiu et al., 2009; Worth et al., 2013), and the current distribution may not have been shaped by northward range expansion such as has occurred for widespread species (Fujii et al., 2002; Magri et al., 2006; Sakaguchi, Takeuchi, Yamasaki, Sakurai, & Isagi, 2011).

## 5 Conclusion

Statistical phylogeographic analyses of *R. weyrichii* populations using model‐based approaches revealed that history of population isolation between islands is likely to have a considerable influence on population survival on each island, even if the islands were sometimes connected geographically during climatic oscillations (Qi et al., 2014). Especially, differences in survival history due to the suitability of isolated habitats are reflected in the current effective population size on each island. The genetic structure of long‐lived woody species would certainly have been influenced by environments during the last glacial.

## Conflict of Interest

None declared.
