# Supplementary material for: Population demographic history of a temperate shrub, Rhododendron weyrichii (Ericaceae), on continental islands of Japan and South Korea
Source: Ecol Evol. 2016 Nov 21;6(24):8800–10. doi: 10.1002/ece3.2576 (PMC5192946; doi:10.1002/ece3.2576)
Supplement: Supplementary file 1 [file ECE3-6-8800-s001.pdf]

Appendix S1 Methods of demographic analysis applied to *Rhododendron weyrichii*

Parental demographic history was inferred using an isolation with migration (IM) model (Hey and Nielsen 2004). The IM model implemented in IMa2 assumes selective neutrality of the loci and no recombination within a locus. To extract recombination-filtered blocks occurring within loci, we used the four-gamete test (Hudson and Kaplan 1985) implemented in IMgc (Woerner et al. 2007). In this analysis, a population splitting model was validated against phylogenetic relationships between four island populations (Jeju, JJ; Kyushu, KY; Shikoku, SK; Kii, KI) based on nuclear DNA sequences (see Figs. S2, 3). The phylogenetic relationships between island populations were reconstructed using the *BEAST option implemented in BEAST v. 1.7.5 (Drummond and Rambaut, 2007; Heled and Drummond 2010). The sequences used in this analysis were the same as those in the IM analysis. Eight individuals from each island population and *R. sanctum* having sequences for all loci were used. The general time-reversible substitution model with discrete gamma-distributed rate variation across sites (GTR+Γ) was used in the analysis. The Yule speciation process, a strict clock model and constant population size were assumed. These models were justified by Bayes factors. Two independent runs were conducted, each of 100 million MCMC steps and the parameters were sampled every 1,000 steps using BEAST with the UPGMA starting tree model. The stationarity and convergence of the parameters after 2 million burn-in periods were checked using Tracer v. 1.6 and two independent runs were combined using LogCombiner v. 1.7.5 (Rambaut et al. 2014). The population splitting model used in IM analysis, which was justified by the phylogenetic relationships, was as follows: the ancestral population (A) of four islands was assumed to split into two descendant populations (1A, ancestral population of JJ and KY; 2A,

ancestral population of SK and KI) at T_{1A+2A} (Fig. 3; Hey 2010). Subsequently, population 1A split into two descendant populations [JJ, KY] at T_{JJ+KY} and population 2A also split into two populations [SK, KI] at T_{SK+KI}. The infinite site (IS) model for nucleotide substitutions was used (Kimura 1969). After several preliminary runs using a wide range of prior settings with different seeds, the prior ranges were set to $t_{JJ+KY} = t_{SK+KI} = t_{1A+2A} = 0.0$ - 0.5, $m = 0$ - 60, $\theta_{JJ} = \theta_{KY} = \theta_{SK} = \theta_{KI} = 0.0$ - 2.0 and $\theta_{1A} = \theta_{2A} = \theta_{A} = 0$ - 15 with a uniform distribution. MCMC simulations were conducted for 5.0×10^7 MCMC steps after a burn-in period of 2.0×10^6 using Metropolis coupling implemented using 50 chains under the two geometric increment model (h1 = 0.90, h2 = 0.60). Finally, with 50 thinning intervals, 10^6 posteriors were sampled and used in calculations of posterior mode, 95% highest posterior density, and the following analyses. To evaluate whether the full IM model fitted better than a series of nested models in which zero migration between populations (m and $2NM = 0$) was assumed, a likelihood ratio test implemented in IMa2 was used. To convert the divergence time to years (T), the geometric mean (2.372×10^{-8}) of the mutation rates per site per generation reported for other plant species [1.50×10^{-8} per site per generation (one year) in *Arabidopsis* (Koch et al. 2000) and 3.75×10^{-8} per site per generation (15 years) in *Populus* (Tuskan et al. 2006; Ingvarsson 2008)] was used, because estimates of the mutation rate per generation vary among taxa. In addition, the mutation rate per year in long-lived species such as trees is considered to be affected by generation time (Petit and Hampe 2006). On the basis of the growth and reproductive characteristics of related species (Morimoto et al. 2003; Yasaka 2006), the maturation of species in *Rhododendron* sect. *Brachycalyx* may require around 15 years, so using 15 years as the generation time, the mutation rate was determined to be 1.581×10^{-9} per site per year. Consequently, a value of $\mu =$

6.236×10^{-7} per year per recombination site-excluded locus was used.

The approximate Bayesian computation (ABC) approach was used to infer changes in the population sizes of island populations by examining four different demographic models (Fig. S3). Model 1, a standard neutral model, has only one parameter: mutation-scaled current effective population size ($\theta_0 = 4N_0\mu$). Model 2, an exponential growth model, has two parameters: θ_0 and constant growth rate (α). Model 3, an instantaneous size reduction model, has three parameters: θ_0, mutation-scaled ancestral population size (θ_A) and the time when the population size changed (t). Model 4, an exponential growth after instantaneous size reduction model (Model 2 + Model 3), has five parameters: θ_0, mutation-scaled reduced population size during the bottleneck (θ_1), mutation-scaled population size before the bottleneck (θ_2), the time at which the population size began to recover from the bottleneck (t_1) and the time at which the population size was reduced at the bottleneck (t_2). The unit of time in models 3 and 4 was generation $\times$ μ. All priors of these four models were uniformly distributed and their ranges were as shown in Figure S4. Prior data sets for the four models were generated using the program R v. 3.2.1 (R Core Team 2015) and coalescent simulations were conducted by the program ms (Hudson 2002). In all simulations, locus-specific θ_0 values were used, which were derived by multiplying the relative sequence length for each locus by the average sequence length among eight loci. When applying ms, time parameters in models 3 and 4 were divided by θ_0 and their units were converted to generation/$4N_0$. The data sets used in this analysis were the same as those in the IM analysis. Coalescent simulations were repeated 1 million times for each of eight loci and the locus-specific summary statistics, nucleotide diversity, θ_W and Kelly's Z_{nS} (Kelly 1997), were calculated using the program msABC (Palvidis et al. 2010). The average and

variance values of the locus-specific summary statistics among eight loci were calculated and finally 1 million summary statistic sets were obtained. Simulated samples were accepted only when they were sufficiently close to the observed data. A tolerance value of 0.001 was used to estimate the posterior distributions of model parameters. Posterior distributions of parameters were estimated by the regression method using neural networks implemented in the abc package of R (Blum and Francois 2012; Csillery et al. 2012). In the process of estimating posterior distributions by the regression method, to prevent the estimated posteriors falling outside the lower or upper bounds of the priors, the logistic option of the abc function was used. The posterior mode and 95% highest posterior density (HPD) were calculated using the coda package in R (Plummer et al. 2006). Candidate models were compared using posterior model probabilities estimated by the multinomial logistic regression approach with a tolerance value of 0.001. Finally posterior predictive simulations were conducted to assess goodness of fit to the data for the parameters estimated from the posterior distributions (Csillery et al. 2012; Gelman et al. 2014). Ten thousand new parameter sets were generated using parameters sampled from posterior distributions. Locus average and variance values for nucleotide diversity, θ_{W} and Kelly's Z_{nS} were calculated from these simulated data sets and compared to the corresponding observed data.

References

Blum MGB, Francois O (2010) Non-linear regression models for Approzimate Bayesian Computation. Stat Compt 20:63-73

Csillery K, Francois O, Blum MGB (2012) abc: an R package for approximate Bayesian computation (ABC). Method Ecol Evol 3: 475–479

Drummond AJ, Rambaut A (2007) BEAST: Bayesian evolutionary analysis by sampling trees. BMC Evol Biol 7: 214.

Gelman A, Carlin JB, Stern HS, Rubinand DB (2014) Bayesian data analysis. Chapman & Hall/CRC Press, Boca Raton

Heled J, Drummond AJ (2010) Bayesian inference of species trees from multilocus data. Mol Biol Evol 27: 570–580

Hey J (2010) Isolation with migration models for more than two populations. Mol Biol Evol 27: 905–920

Hey J, Nielsen R (2004) Multilocus methods for estimating population sizes, migration rates and divergence time, with applications to the divergence of *Drosophila pseudoobscura* and *D. persimilis*. Genetics 167: 747–760

Hudson RR (2002) Generating samples under a Wright-Fisher neutral model. Bioinformatics 18: 337–338

Hudson RR, Kaplan NL (1985) Statistical properties of the number of recombination events in the history of a sample of DNA sequences. Genetics 111: 147–164

Ingvarsson PK (2008) Multilocus patterns of nucleotide polymorphism and the demographic history of *Populus tremula*. Genetics 180: 329-340

Kelly JK (1997) A test of neutrality based on interlocus associations. Genetics 146:1197-1206

Kimura M (1969) The number of heterozygous nucleotide sites maintained in a finite population due to steady flux of mutations. Genetics 61: 893–903

Koch MA, Haubold B, Mitchell-Olds T (2000) Comparative evolutionary analysis of chalcone synthase and alcohol dehydrogenase loci in *Arabidopsis*, *Arabis*, and related genera (Brassicaceae). Mol Biol Evol 17: 1483–1498

Morimoto J, Shibata S, Hasegawa S (2003) Habitat requirement of *Rhododendron reticulatum* and *R. macrosepalum* in germination and seedling stages- field experiment for restoration of native *Rhododendron* by seeding. J Jap Soc Reveg Tech 29: 135–140. (in Japanese)

Pavlidis P, Laurent S, Stephan W (2010) msABC: a modification of Hudson's ms to facilitate multi-locus ABC analysis. Mol Ecol Res 10:723-727

Petit RJ, Hampe A (2006) Some evolutionary consequences of being a tree. Annu Rev Ecol

Evol Syst 37: 187–214

Plummer M, Best N, Cowles K, Vines K (2006) CODA: Convergence Diagnosis and Output Analysis for MCMC. R News 6:7-11

R Development Core Team (2015) R: A language and environment for statistical computing.R Foundation for Statistical Computing, Vienna

Rambaut A, Suchard MA, Xie D, Drummond AJ (2014) Tracer v1.6, Available from http://beast.bio.ed.ac.uk/Tracer

Tuskan GA, DiFazio S, Jansson S, Bohlmann J, Grigoriev I, Hellsten U, Putnam N, Ralph S, Rombauts S, Salamov A, Schein J, Sterck L, Aerts A, Bhalerao RR, Bhalerao RP, Blaudez D, Boerjan W, Chen G-L, Cooper D, Coutinho PM, Couturier J, Covert S, Cronk Q, Cunningham R, Davis J, Degroeve S (2006) The genome of black cottonwood, *Populus trichocarpa* (Torr. & Gray). Science 313: 1596–1604

Woerner AE, Cox MP, Hammer MF (2007) Recombination-filtered genomic datasets by information maximization. Bioinformatics 23: 1851–1853

Yasaka M (2006) For conserving an endangered species, *Rhododendron dilatatum* var. *boreale*. Kousyunaikihou 143: 18–22. (in Japanese)

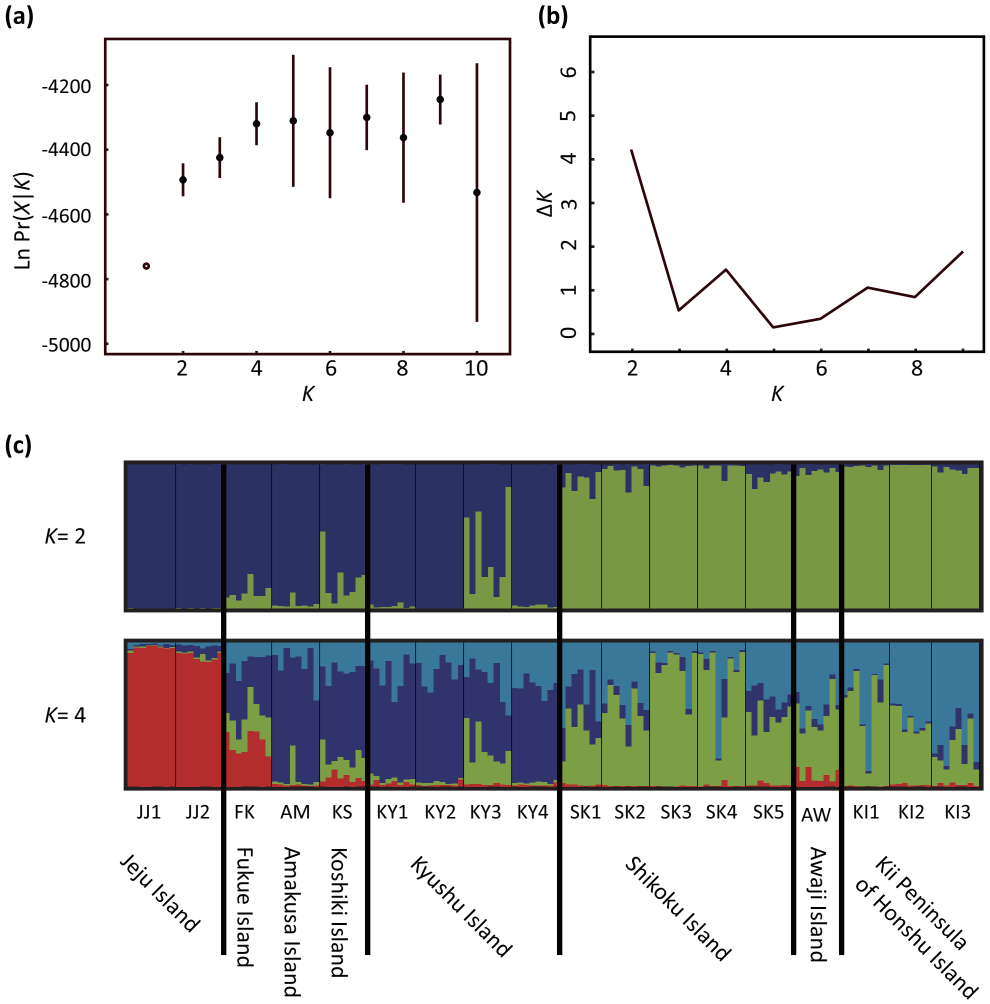

Fig. S1. (a) Log-likelihood of the data (Ln Pr(X | K)) for 10 independent runs and (b) second order rates of change in log-likelihood, ΔK, as a function of the number of clusters. (c) Distribution of clusters across individuals from the 18 populations, with the optimal numbers of clusters (K), based on ΔK and log-likelihood, being 2 and 4 respectively.

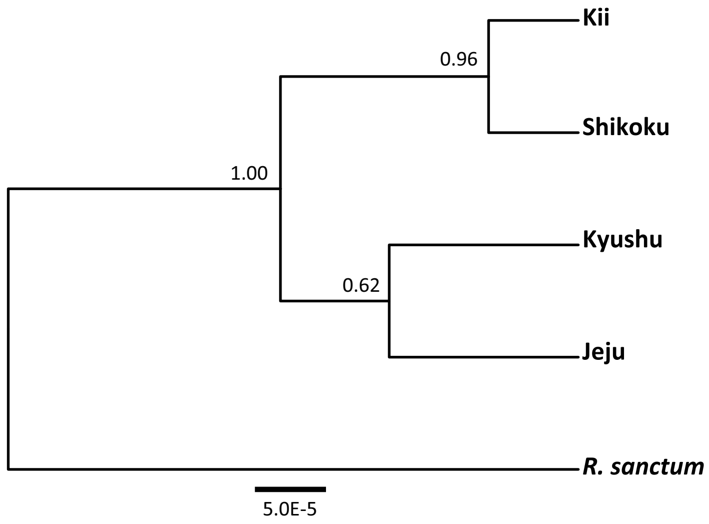

Fig. S2. Phylogenetic relationships between island populations and an outgroup species based on eight nuclear DNA loci and determined by the Bayesian method. Posterior probabilities are shown above nodes.

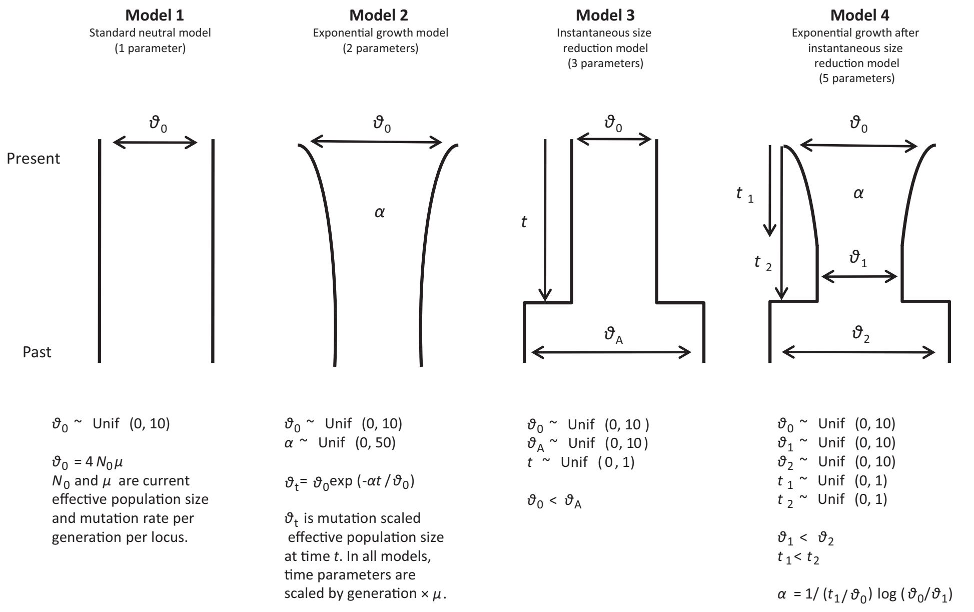

Fig. S3. The four population demographic models examined in this study and their prior distributions. Model 1, standard neutral model; Model 2, exponential growth model; Model 3, instantaneous size reduction model; Model 4 exponential growth after instantaneous size reduction model.

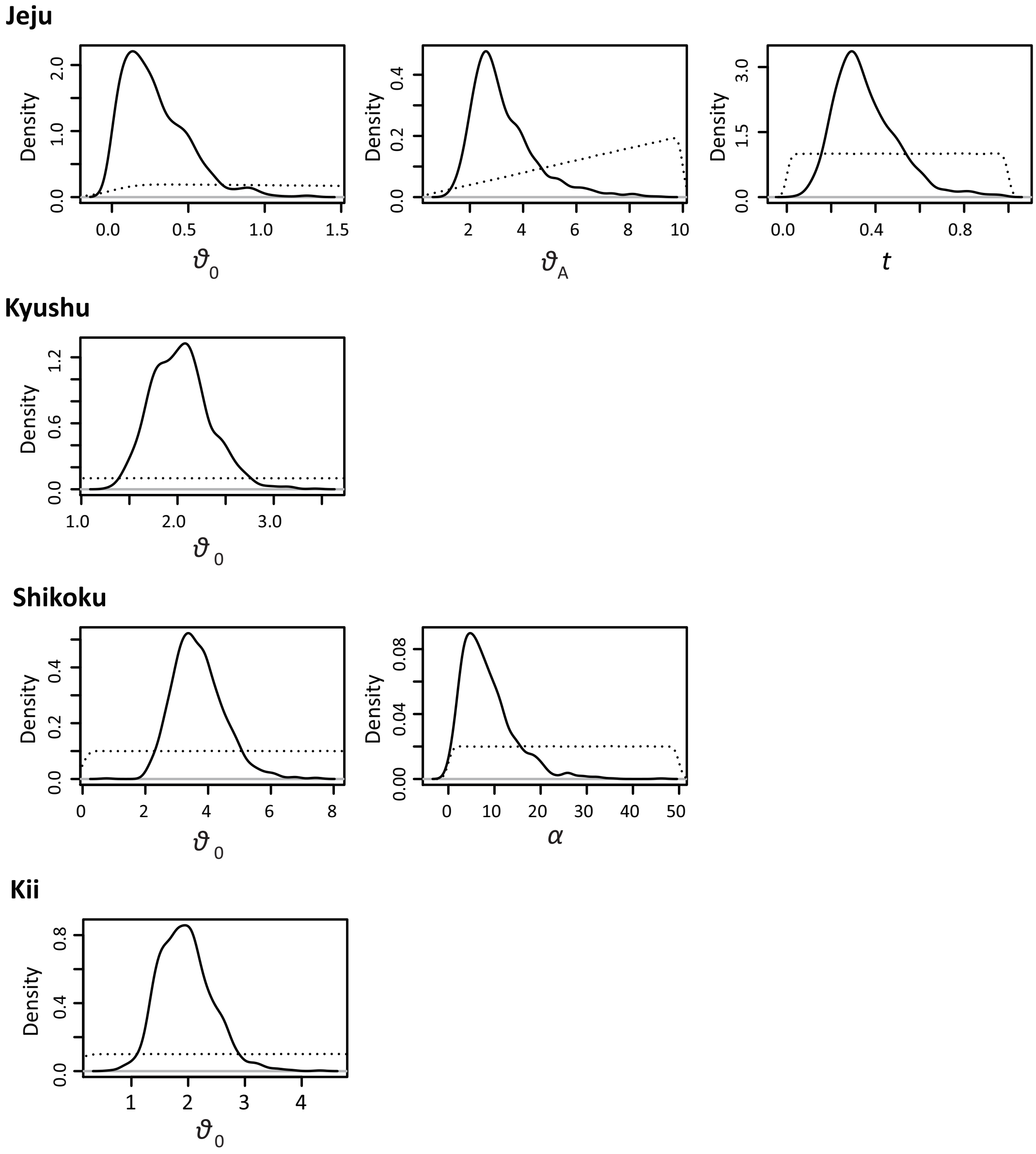

Fig. S4. Posterior distributions of demographic parameters in the models accepted by the approximate Bayesian computation (ABC) approach for the populations on four continental islands. Dotted lines indicate prior distributions.

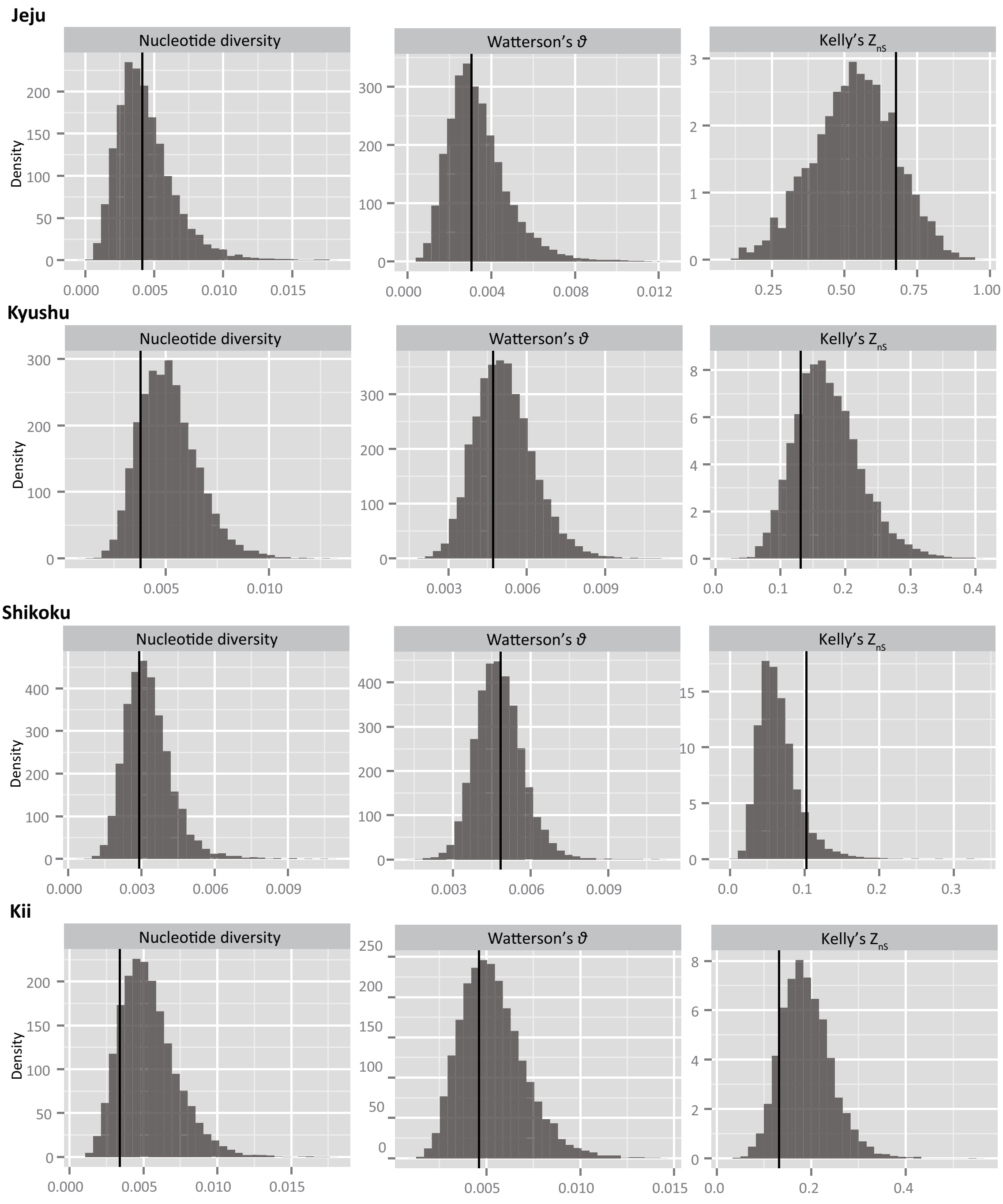

Fig. S5. Posterior prediction of average and variance for summary statistics (π, nucleotide diversity; θ_W, Watterson's theta; Z_{nS}, Kelley's Z_{nS}) using posterior distribution of accepted models for each island. Vertical lines indicate the observed values.

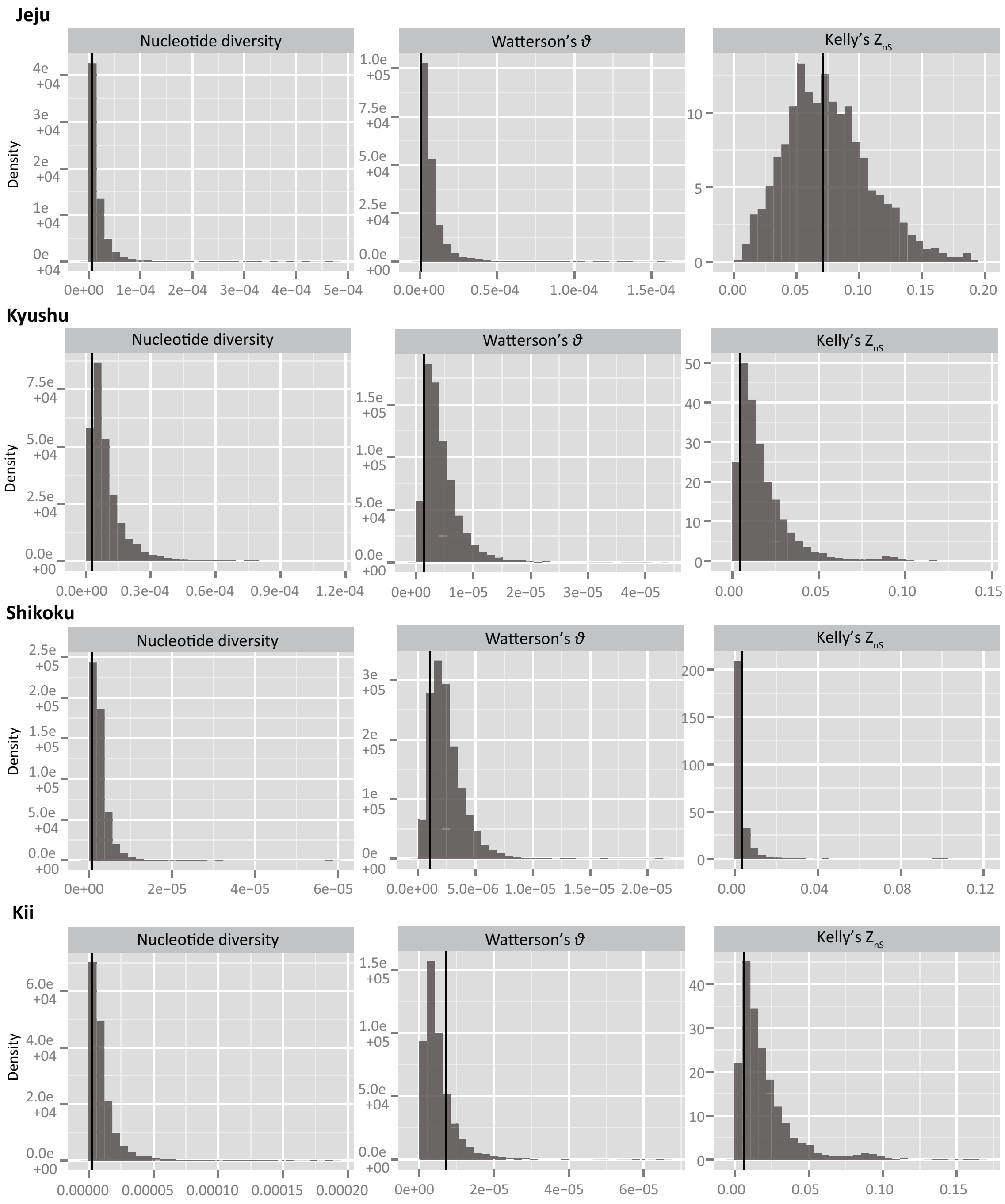

Fig. S5. Continued.

Table S1. Putative orthologs identified by homology searches against the NCBI and *Arabidopsis thaliana* (TAIR) polypeptide databases for the eight nuclear DNA loci

Locus	Gene	Function	*E*-value (NCBI)	*E*-value (TAIR)	Primer source
EST39	*ASN1*	Glutamine-dependent asparagine synthetase	3e-36 (*Ricinus communis*)	5e-34	De Keyser et al. (2009)
EST65	*KING1*	SNF1-related protein kinase regulatory subunit gamma	4e-41 (*Rhododendron mariesii*)	4e-22	De Keyser et al. (2009)
EST121	*ATHDH*	Histidinol dehydrogenase	2e-14 (*Vitis vinifera*)	3e-11	De Keyser et al. (2009)
EST136	-	-	-	-	De Keyser et al. (2009)
C16	-	-	-	-	Wei et al. (2005)
C22	*CAB4*	Chlorophyll a/b-binding protein	1e-54 (*Musa acuminata*)	3e-46	Wei et al. (2005)
PHYB	*PHYB*	Phytochrome defective	3e-105 (*Phyllodoce nipponica*)	4e-76	Ikeda & Setoguchi (2010)
PHYE	*PHYE*	Phytochrome defective	2e-58 (*Kalmiopsis fragrans*)	5e-56	Ikeda & Setoguchi (2010)

Names and functions of candidate genes with low *E* value (E < 1e-10) are shown

Table S2. Pairwise estimate of genetic differentiation (average number of nucleotide differences, D_{xy}) between populations over eight nuclear loci

		JJ1	JJ2	FK	AM	KS	KY1	KY2	KY3	KY4	SK1	SK2	SK3	SK4	SK5	AW	KI1	KI2	KI3
Jeju	JJ1	0.0000	0.0048	0.0058	0.0059	0.0061	0.0060	0.0062	0.0059	0.0069	0.0059	0.0063	0.0058	0.0064	0.0054	0.0059	0.0061	0.0062	0.0062
	JJ2	0.0048	0.0000	0.0045	0.0043	0.0045	0.0045	0.0047	0.0045	0.0052	0.0042	0.0048	0.0042	0.0047	0.0037	0.0045	0.0046	0.0046	0.0048
Fukue	FK	0.0058	0.0045	0.0000	0.0044	0.0044	0.0047	0.0047	0.0044	0.0054	0.0043	0.0047	0.0040	0.0047	0.0039	0.0046	0.0046	0.0046	0.0051
Amakusa	AM	0.0059	0.0043	0.0044	0.0000	0.0044	0.0045	0.0044	0.0043	0.0052	0.0042	0.0047	0.0041	0.0048	0.0037	0.0046	0.0047	0.0046	0.0049
Koshiki	KS	0.0061	0.0045	0.0044	0.0044	0.0000	0.0047	0.0048	0.0045	0.0055	0.0044	0.0047	0.0040	0.0046	0.0039	0.0045	0.0046	0.0045	0.0048
Kyushu	KY1	0.0060	0.0045	0.0047	0.0045	0.0047	0.0000	0.0046	0.0045	0.0053	0.0043	0.0047	0.0042	0.0049	0.0040	0.0048	0.0049	0.0048	0.0049
	KY2	0.0062	0.0047	0.0047	0.0044	0.0048	0.0046	0.0000	0.0045	0.0052	0.0044	0.0049	0.0044	0.0052	0.0041	0.0050	0.0050	0.0050	0.0051
	KY3	0.0059	0.0045	0.0044	0.0043	0.0045	0.0045	0.0045	0.0000	0.0053	0.0041	0.0045	0.0037	0.0045	0.0037	0.0044	0.0044	0.0044	0.0048
	KY4	0.0069	0.0052	0.0054	0.0052	0.0055	0.0053	0.0052	0.0053	0.0000	0.0053	0.0057	0.0052	0.0057	0.0050	0.0058	0.0059	0.0057	0.0060
Shikoku	SK1	0.0059	0.0042	0.0043	0.0042	0.0044	0.0043	0.0044	0.0041	0.0053	0.0000	0.0040	0.0032	0.0041	0.0031	0.0039	0.0040	0.0039	0.0043
	SK2	0.0063	0.0048	0.0047	0.0047	0.0047	0.0047	0.0049	0.0045	0.0057	0.0040	0.0000	0.0036	0.0045	0.0036	0.0045	0.0043	0.0043	0.0046
	SK3	0.0058	0.0042	0.0040	0.0041	0.0040	0.0042	0.0044	0.0037	0.0052	0.0032	0.0036	0.0000	0.0032	0.0027	0.0035	0.0032	0.0033	0.0038
	SK4	0.0064	0.0047	0.0047	0.0048	0.0046	0.0049	0.0052	0.0045	0.0057	0.0041	0.0045	0.0032	0.0000	0.0036	0.0041	0.0040	0.0039	0.0046
	SK5	0.0054	0.0037	0.0039	0.0037	0.0039	0.0040	0.0041	0.0037	0.0050	0.0031	0.0036	0.0027	0.0036	0.0000	0.0034	0.0034	0.0033	0.0038
Awaji	AW	0.0059	0.0045	0.0046	0.0046	0.0045	0.0048	0.0050	0.0044	0.0058	0.0039	0.0045	0.0035	0.0041	0.0034	0.0000	0.0041	0.0040	0.0046
Kii	KI1	0.0061	0.0046	0.0046	0.0047	0.0046	0.0049	0.0050	0.0044	0.0059	0.0040	0.0043	0.0032	0.0040	0.0034	0.0041	0.0000	0.0039	0.0044
	KI2	0.0062	0.0046	0.0046	0.0046	0.0045	0.0048	0.0050	0.0044	0.0057	0.0039	0.0043	0.0033	0.0039	0.0033	0.0040	0.0039	0.0000	0.0040
	KI3	0.0062	0.0048	0.0051	0.0049	0.0048	0.0049	0.0051	0.0048	0.0060	0.0043	0.0046	0.0038	0.0046	0.0038	0.0046	0.0044	0.0040	0.0000

Table S3. Modes and 95% highest posterior density (HPD) intervals for (a) split time (t) and scaled divergence time (T), (b) population size at present time (θ), (c) migration rate per mutation event (m) and (d) population migration rate ($2NM = 0.5\theta m$) for the populations on four continental islands (Kii, Shikoku, Kyushu and Jeju) and their ancestral populations estimated using the isolation with migration (IM) model

(a)		t_{JJ+KY}	t_{SK+KI}	t_{1A+2A}	T_{JJ+KY}	T_{SK+KI}	T_{1A+2A}
Mode		0.043	0.006	0.151	68,554	9,221	242,623
95% HPD	Lower	0.008	0.001	0.051	13,230	2,004	81,302
	Upper	0.276	0.041	0.384	443,072	65,346	616,100

(b)		θ_{JJ}	θ_{KY}	θ_{SK}	θ_{KI}	θ_{1A}	θ_{2A}	θ_{A}
Mode		0.099	0.415	0.515	0.239	2.228	5.152	1.718
95% HPD	Lower	0.025	0.183	0.083	0.047	0.293	3.007	0.953
	Upper	0.291	1.243	1.189	0.647	14.29	14.62	2.678

(c)		$m_{JJ>KY}$		$m_{KY>JJ}$		$m_{KY>SK}$		$m_{SK>KY}$		$m_{SK>KI}$		$m_{KI>SK}$		$m_{1A>2A}$		$m_{2A>1A}$	
Mode [a]		10.17	*	0.03	ns	26.79	*	6.99	ns	0.03	ns	27.09	ns	3.21	ns	1.41	ns
95% HPD	Lower	0.00		0.00		3.99		0		0		4.89		0		0	
	Upper	38.61		8.73		58.41		41.13		41.91		59.97		55.83		55.17	

(d)		$2N_{JJ}M_{JJ>KY}$		$2N_{KY}M_{KY>JJ}$		$2N_{KY}M_{KY>SK}$		$2N_{SK}M_{SK>KY}$		$2N_{SK}M_{SK>KI}$		$2N_{KI}M_{KI>SK}$		$2N_{1A}M_{1A>2A}$		$2N_{2A}M_{2A>1A}$	
Mode [a]		0.7688	*	0.006165	ns	6.627	**	2.511	ns	1.508	ns	4.296	ns	3.823	ns	3.823	ns
95% HPD	Lower	0		0		0		0		0		0		0		0	
	Upper	1.641		2.349		15.26		6.236		6.868		8.413		292.6		287.6	

JJ, Jeju; KY, Kyushu; SK, Shikoku; KI, Kii; 1A, ancestral population of Jeju and Kyushu populations; 2A, ancestral population of Shikoku and Kii

populations; A, ancestral population of all island populations. Units of the t and T are generation × μ and years, respectively. The directions of migrations indicated by the subscripts of m and 2NM were defined in the context of the coalescent. For example, $m_{B>C}$ indicates that genes move from C to B in the sense of time in the forward direction.

[a] Significances of migration parameters (m and 2NM) when compared with the null model were tested by likelihood ratio tests. * $P < 0.05$; ns, not significant.

Table S4. Posterior modes and 95% highest posterior density (HPD) intervals of parameters in the models accepted by the approximate Bayesian computation (ABC) approach for the populations on four continental islands

Population	Accepted model[a]	Parameter	Mode	95% HPD	
				Lower	Upper
Jeju	Model 3	θ_0	0.136	0.000	0.673
		θ_A	2.612	1.391	5.768
		t (generation×μ)	0.292	0.115	0.642
		T (kilo years ago)	467,009	183,950	1,025,729
Kyushu	Model 1	θ_0	2.076	1.441	2.603
Shikoku	Model 2	θ_0	3.365	2.168	5.178
		α	4.775	0.008	19.097
Kii	Model 1	θ_0	1.942	1.184	2.930

[a] Model 1, standard neutral model; Model 2, exponential growth model; Model 3, instantaneous size reduction model; Model 4, exponential growth after instantaneous size reduction model.

[b] T (year) was estimated assuming that generation time was 15 years.

Tolerance was set to 0.001.
